# 24-h dietary intake and its relationship with nutritional knowledge and behaviors in older adults with type 2 diabetes in Vietnam

**DOI:** 10.3389/fnut.2025.1602979

**Published:** 2025-09-12

**Authors:** Duong Thuy Thi Truong, Hoa Thi Nguyen, Hoa Thanh Thi Le

**Affiliations:** ^1^Department of Nutrition and Food Safety, Thai Nguyen University of Medicine and Pharmacy, Thai Nguyen University, Thai Nguyen, Vietnam; ^2^Vinh Yen City Medical Center, Vinh Phuc, Vietnam; ^3^Department of Environmental Health and Occupational Health, Thai Nguyen University of Medicine and Pharmacy, Thai Nguyen University, Thai Nguyen, Vietnam

**Keywords:** type 2 diabetes, dietary intake, nutritional knowledge, dietary behavior, older adults, Vietnam, glycemic control, 24-h dietary recall

## Abstract

**Background:**

Type 2 diabetes mellitus (T2DM) is a growing public health challenge in Vietnam, particularly among older adults, with dietary intake critical for its management. Limited research explores how nutritional knowledge and dietary behaviors influence dietary intake in Vietnamese older adults with T2DM.

**Objective:**

This study assesses 24-h dietary intake and its relationship with nutritional knowledge and dietary behaviors in older adults with T2DM in Vietnam.

**Methods:**

A cross-sectional study was conducted among 355 older adults with T2DM at Vinh Yen City Medical Center, Vinh Phuc, Vietnam. Data included anthropometric measurements, biochemical parameters, and 24-h dietary recall. Nutritional knowledge and dietary behaviors were assessed via structured questionnaires. Regression analyses examined associations with glycemic control.

**Results:**

Overweight/obese participants had significantly higher energy (1,331.1 kcal/day vs. 1,104.9 kcal/day, *P* < 0.001), protein (74.2 g/day vs. 59.9 g/day, *P* < 0.001), carbohydrate (199.2 g/day vs. 168.9 g/day, *P* = 0.022), and fat intake (26.4 g/day vs. 21.1 g/day, *P* = 0.034) than normal-weight participants. Poor nutritional knowledge was prevalent in 51% of overweight/obese vs. 19% of normal-weight participants (*P* < 0.001), with only 16.3% adhering to dietary guidelines. Higher energy intake was associated with increased HbA1c (β = 0.15, *P* = 0.049), while carbohydrate intake showed an inverse relationship (β = −0.60, *P* = 0.049). Higher BMI was linked to lower HbA1c (β = −0.15, *P* = 0.029).

**Conclusion:**

Older adults with T2DM in Vietnam show significant variations in dietary intake, with poor nutritional knowledge, and low dietary adherence, particularly among overweight/obese individuals. Poor dietary adherence and limited nutritional understanding, particularly among overweight/obese individuals, highlight the need for targeted dietary interventions. Structured nutritional counseling and culturally tailored education programs may improve adherence and glycemic control in this population. Interventions are needed to address barriers like limited knowledge and economic constraints, thereby improving glycemic control and informing public health policies.

## 1 Introduction

Type 2 diabetes mellitus (T2DM) is a significant public health concern in Vietnam, with rising prevalence, particularly among older adults due to rapid urbanization, dietary transitions, and lifestyle changes (1). Older adults with T2DM face unique barriers to effective management, including limited access to nutritional education, economic constraints, reduced appetite due to age-related comorbidities, and difficulty integrating dietary changes (2). Many T2DM patients have poor glycemic control and experience complications like neuropathy and heart disease, emphasizing the need for comprehensive strategies focused on dietary intake and nutritional behaviors (3). Dietary patterns high in insulinemic and inflammatory potential exacerbate T2DM risk and control in Vietnamese populations (4).

Despite the known benefits of dietary interventions, adherence to nutritional recommendations remains suboptimal. In Vietnam, few T2DM patients adhere to dietary guidelines, with barriers including limited knowledge and economic constraints (5). Structured dietary management behaviors improve diet quality, as assessed by indices like the China Healthy Diet Index (CHDI), which evaluates components such as fruits, vegetables, whole grains, and sodium (6). The American Diabetes Association (ADA) emphasizes diverse nutrient intake for T2DM management (7). Social and behavioral factors, including support from families and health professionals, significantly impact adherence, but knowledge gaps and integration difficulties persist (8).

Despite these insights, there remains a gap in understanding the relationship between actual dietary intake and nutritional knowledge and behaviors among older adults with T2DM in Vietnam. Few studies have assessed how dietary intake aligns with knowledge and adherence to recommended practices, or how social factors influence choices in this population. This study aims to assess 24-h dietary intake and its relationship with nutritional knowledge and behaviors in older adults with T2DM. By integrating quantitative and qualitative approaches, it analyzes dietary patterns, adherence, and influencing factors. The findings will inform culturally tailored interventions to improve diabetes management, addressing the rising prevalence and critical role of diet in health outcomes for older Vietnamese adults with T2DM.

## 2 Materials and methods

### 2.1 Study design

This study employs a cross-sectional descriptive design to assess 24-h dietary intake and its relationship with nutritional knowledge and behaviors among patients with type 2 diabetes mellitus in Vietnam. A cross-sectional approach was chosen as it allows for efficient data collection and provides a snapshot of dietary behaviors and knowledge at a specific time.

### 2.2 Study population

This cross-sectional study included 355 older adults (mean age 65.5 years, SD 10.2) diagnosed with T2DM per WHO criteria, receiving care at Vinh Yen City Medical Center, Vinh Phuc, Vietnam.

#### 2.2.1 Inclusion criteria

- Patients diagnosed with type 2 diabetes mellitus per WHO criteria (9).
- Patients receiving care and treatment at Vinh Yen City Medical Center, Vinh Phuc, Vietnam.
- Patients who provided written informed consent to participate.
- Adults aged ≥ 50 years to focus on older adults, reflecting the demographic skew in our sample.

#### 2.2.2 Exclusion criteria

- Patients diagnosed with type 1 diabetes or gestational diabetes mellitus (GDM).
- Patients with cognitive impairments or communication difficulties prevent interview participation.
- Patients with malabsorption disorders or gastrointestinal conditions that significantly alter dietary intake, to ensure accurate recall data (4).
- Prior nutrition education was not an exclusion criterion, as it reflects real-world patient diversity, though not systematically recorded, aligning with studies like Tran et al. (2) that note variable education exposure.

### 2.3 Study setting and duration

- Study period: December 2022–October 2023.
- Study location: Vinh Yen City Medical Center, Vinh Phuc, Vietnam, is a public healthcare facility providing diabetes management and treatment services.

### 2.4 Sample size and sampling method

#### 2.4.1 Sample size calculation

A total of 355 T2DM patients were included, representing the entire population of eligible patients at the study site. Given the census-based approach, no formal sample size calculation was required.

#### 2.4.2 Sampling strategy

A purposive sampling method was used to recruit all eligible T2DM patients managed at the medical center during the study period. This approach ensured maximum data representativeness by including all available patients who met the selection criteria.

### 2.5 Data collection procedures

#### 2.5.1 Anthropometric measurements

Standardized procedures were used to assess body composition and anthropometric indicators (10):

- Weight: Measured using a SECA electronic scale (accuracy: ±0.1 kg), with participants wearing light clothing and no shoes.
- Height: Measured with a UNICEF stadiometer (precision: ±1 mm), ensuring proper posture.
- Waist circumference: Measured at the midpoint between the iliac crest and the lower rib margin using a non-stretchable tape measure.
- Hip circumference: Measured at the widest part of the buttocks with a flexible tape.

Body mass index (BMI): Calculated as weight (kg)/height^2^ (m^2^) and classified according to (11) BMI cut-off points as low risk (18.5–24.9 kg/m^2^) and high risk (≥ 25 kg/m^2^ for overweight/obese).

#### 2.5.2 Dietary intake assessment

A 24-h dietary recall (24HR) was conducted to assess food and nutrient intake. Trained investigators collected nutritional data using a standardized structured questionnaire. The multi-pass method was used to improve recall accuracy, as validated by Moshfegh et al. (12) for reducing energy intake bias:

- Quick list: Participants list all foods and beverages consumed in the previous 24 h.
- Detailed description: Additional information on portion sizes, preparation methods, and brand names.
- Review: Final verification for completeness and accuracy. Portion sizes were estimated using food models and household utensils commonly used in Vietnam. Nutrient intake was analyzed using Vietnamese food composition tables.

#### 2.5.3 Nutritional knowledge and dietary behavior assessment

- Nutritional knowledge: Assessed using a 9-item questionnaire based on national and international T2DM dietary guidelines, categorized as “Good” (≥ 70% correct) or “Poor” (< 70% correct).
- Dietary behavior: Measured using a 10-item questionnaire based on national and international dietary guidelines, categorizing responses as “Good” (≥ 70% adherence) or “Poor” (< 70% adherence).

The questionnaire was not formally validated but underwent expert review for content validity.

#### 2.5.4 Biochemical data collection

Biochemical parameters were retrieved from medical records, with IRB approval (No: 1283/DHYD-HĐĐD) covering chart review (3). Tests followed standardized protocols (9). Blood samples (5 mL) were collected after ≥ 8 h fasting; new tests were conducted if recent data were unavailable (1).

- Fasting blood glucose (mmol/L)
- Glycated hemoglobin (HbA1c) (%)
- Triglycerides (mmol/L)
- Total cholesterol (mmol/L).
- The cut-off values for biochemical control were based on the (13).

### 2.6 Data analysis and missing data handling

Data were analyzed using descriptive statistics for dietary intake and nutritional status, *t*-tests or ANOVA for group comparisons, and multivariable logistic regression for associations, adjusting for age, gender, education, and income. All analyses were performed with SPSS version 26, with significance at *P* < 0.05.

Missing data for key variables were minimal and handled appropriately to minimize bias. Anthropometric measurements had < 2% missing (*n* = 6/355, 1.7%), primarily due to participant refusal; these were excluded pairwise. Dietary recall data had < 2% missing (*n* = 5/355, 1.4%), due to incomplete responses, and were also excluded pairwise. Nutritional knowledge and behavior questionnaires had 0% missing, as they were interviewer-administered. Biochemical parameters (e.g., HbA1c) had 4.2% missing (*n* = 15/355), due to lab processing errors. Multiple imputation was used only for biochemical parameters where missing exceeded 5%, employing predictive mean matching with 5 iterations and 5 imputed datasets, pooled via Rubin’s rules. This method was chosen for its robustness in handling missing at random (MAR) data, as confirmed by Little’s MCAR test (*P* = 0.12). Sensitivity analyses with complete cases yielded similar results.

## 3 Ethical considerations

This study received ethical approval from the Ethics Committee of the Thai Nguyen University of Medicine and Pharmacy (Approval No: 1283/ĐHYD-HĐĐĐ on 28th December 2022). Written informed consent was obtained from all participants before data collection, ensuring voluntary participation. Confidentiality and privacy were maintained by anonymizing participant information, securing data in a password-protected database, and restricting access to authorized researchers only. All study procedures complied with the Declaration of Helsinki (2013) and relevant national ethical guidelines. Participants were informed about their right to withdraw without consequences, and no financial incentives were provided for participation.

## 4 Results

Descriptive characteristics of the study population stratified by nutrition status (Table 1). Values are presented as Mean (SD) for continuous variables and *n* (%) for categorical variables. Most participants were classified as normal weight (57.4%, *N* = 202) or overweight/obese (41.8%, *N* = 147), with only 1.7% (*N* = 6) underweight. Gender differences were significant (*P* < 0.001), with 65% of overweight/obese participants male vs. 35% in the normal group. Education level varied (*P* = 0.015), with 29% of overweight/obese and 17% of normal-weight participants university-educated, vs. 0% in underweight. Underweight participants had the highest mean age (72.5 ± 13.6 years) and longest diabetes duration (6.8 ± 4.1 years, *P* < 0.001). Older age in the underweight group may reflect reduced appetite or comorbidities, potentially lowering energy intake (1,180.2 kcal/day). Mean glucose levels were significantly higher in the overweight/obese group (7.8 mmol/L, *P* < 0.001), while HbA1c levels did not differ significantly across groups (*P* = 0.300).

**TABLE 1:** Descriptive characteristics of the study population.

Variable	Category	Underweight (*N* = 6)	Normal (*N* = 202)	Overweight/obese (*N* = 147)	*p*-value
		*n* (%)			
Age (years)	(Mean ± SD)	72.5 (13.6)	64.6 (11.5)	63.9 (12.4)	0.200^1^
Gender	Male	1 (17%)	71 (35%)	95 (65%)	< 0.001^2^
	Female	5 (83%)	131 (65%)	52 (35%)	
Education level	Primary	1 (17%)	2 (1.1%)	2 (1.6%)	0.015^2^
	Secondary	4 (67%)	74 (41%)	45 (35%)	
	High school	1 (17%)	75 (41%)	44 (34%)	
	University/postgrad	0 (0%)	30 (17%)	37 (29%)	
Income level	< 5 million VND	1 (33%)	15 (25%)	4 (17%)	0.600^2^
	≥ 5 million VND	2 (67%)	45 (75%)	19 (83%)	
Years with diabetes		6.8 (4.1)	4.3 (2.8)	2.8 (1.7)	< 0.001^1^
Glucose (mmol/L)		7.3 (1.2)	7.1 (1.5)	7.8 (1.9)	< 0.001^1^
HbA1c		7.7 (1.6)	8.1 (1.3)	7.9 (1.2)	0.300^1^

^1^Kruskal–Wallis rank sum test for continuous variables.

^2^Fisher’s exact test for categorical variables. Continuous variables are presented as mean ± standard deviation (SD). Categorical variables as *n* (%).

Dietary intake characteristics of the study population stratified by nutritional status (Table 2). Values are presented as mean (SD) for continuous nutritional intake variables. Energy intake was significantly higher in the overweight/obese group (1,331.1 kcal/day), followed by the underweight group (1,180.2 kcal/day) and the normal-weight group (1,104.9 kcal/day; *P* < 0.001). Similar trends were observed for protein (*P* < 0.001), carbohydrate (*P* = 0.022), and total fat intake (*P* = 0.034), with overweight/obese participants consuming the highest levels. Saturated fat (*P* = 0.048), total sugars (*P* = 0.032), and glycemic load (*P* = 0.029) were also significantly elevated in the overweight/obese group. Fiber intake differences approached significance (*P* = 0.051), while cholesterol, vitamin A, and vitamin C intake did not significantly differ across groups (all *P* > 0.05). No significant differences were observed for monounsaturated fat, glycemic index, or vitamin D intake.

**TABLE 2:** Dietary intake by nutrition status.

Dietary intake	Underweight (*N* = 6)	Normal (*N* = 202)	Overweight/obese (*N* = 147)	*p*-value^1^
	(Mean ± SD)			
Energy (kcal/day)	1,180.2 (439.1)	1,104.9 (492.1)	1,331.1 (597.9)	< 0.001
Protein (g/day)	58.0 (18.2)	59.9 (22.7)	74.2 (32.8)	< 0.001
Carbohydrates (g/day)	185.7 (100.4)	168.9 (94.0)	199.2 (108.2)	0.022
Total fat (g/day)	23.1 (9.9)	21.1 (19.5)	26.4 (18.2)	0.034
Saturated fat (g/day)	14.8 (6.3)	13.5 (12.4)	16.8 (11.6)	0.048
Monounsaturated fat (g/day)	5.0 (2.1)	4.6 (4.2)	5.7 (3.9)	0.055
Fiber (g/day)	5.4 (1.8)	6.0 (2.5)	7.1 (5.6)	0.051
Cholesterol (mg/day)	3.8 (1.8)	4.1 (1.3)	4.3 (1.3)	0.300
Total sugars (g/day)	24.9 (11.8)	22.5 (14.0)	27.8 (16.3)	0.032
Glycemic index	54.8 (7.9)	54.4 (7.7)	55.9 (8.2)	0.350
Vitamin A (μg/day)	861.6 (1,309.7)	410.6 (1,239.5)	758.7 (1,881.7)	0.100
Vitamin D (μg/day)	4.0 (2.0)	3.7 (1.9)	4.3 (2.2)	0.170
Vitamin C (mg/day)	123.2 (72.7)	119.4 (74.7)	126.0 (83.9)	0.700

^1^One-way ANOVA.

Nutritional knowledge and dietary behavior of the study population stratified by nutritional status (Table 3). Values are presented as *n* (%) for categorical variables. A significant association was observed between nutritional knowledge and nutritional status (*P* < 0.001), with poor nutritional knowledge reported by 51% of overweight/obese participants, compared to 19% in the normal-weight group and 17% in the underweight group.

**TABLE 3:** Nutritional knowledge and behavior by nutrition status.

Variable	Category	Underweight (*N* = 6)	Normal (*N* = 202)	Overweight/obese (*N* = 147)	*p*-value
Nutritional knowledge	Poor	1 (17%)	38 (19%)	75 (51%)	< 0.001^1^
	Good	5 (83%)	164 (81%)	72 (49%)	
Dietary behavior	Poor	1 (17%)	38 (19%)	75 (51%)	< 0.001^1^
	Good	5 (83%)	164 (81%)	72 (49%)	

^1^Pearson’s chi-squared test.

Dietary behavior followed a similar pattern (*P* < 0.001), with poor nutritional behavior observed in 51% of overweight/obese participants, compared to 19% and 17% in the normal-weight and underweight groups, respectively.

Association between dietary intake and clinical outcomes, presented as β [95% confidence intervals (CI)], *p*-values using multiple linear regression (Table 4). Energy intake was significantly associated with higher HbA1c levels (β = 0.15; 95% CI: 0.00, 0.30; *P* = 0.049), while carbohydrate intake showed a significant inverse association with HbA1c (β = −0.60; 95% CI: −1.20, 0.00; *P* = 0.049). Protein and fat intake were marginally associated with lower HbA1c, with protein at *P* = 0.057 and total fat at *P* = 0.051, suggesting possible trends toward glycemic benefit. However, none of the dietary intake variables were significantly associated with fasting glucose levels (all *p* > 0.05).

**TABLE 4:** Association between dietary intake and clinical outcomes.

Characteristic	Glucose (mmol/L) (β, 95% CI)	*p*-value	HbA1c (%) (β, 95% CI)	*p*-value
Energy intake (kcal/day)	0.03 (−0.13, 0.18)	0.700	0.15 (0.00, 0.30)	0.049
Protein intake (g/day)	−0.09 (−0.71, 0.53)	0.800	−0.58 (−1.2, 0.02)	0.057
Carbohydrates intake (g/day)	−0.11 (−0.73, 0.51)	0.700	−0.60 (−1.2, 0.00)	0.049
Total Fat intake (g/day)	−0.23 (−1.6, 1.2)	0.700	−1.3 (−2.7, 0.01)	0.051
Fiber intake (g/day)	0.03 (−0.11, 0.16)	0.700	−0.05 (−0.18, 0.08)	0.400
Age (years)	0.01 (−0.02, 0.04)	0.400	0.03 (0.00, 0.06)	0.041
**Gender (reference: male)**	1	–	–	–
Female	−0.31 (−1.1, 0.44)	0.400	−0.23 (−0.96, 0.51)	0.500
**Income (reference: < 5 million VND)**	1	–	–	–
≥ 5 million VND	0.01 (−0.72, 0.74)	0.900	−0.08 (−0.79, 0.62)	0.800
**Education (reference: primary)**	1	–	–	–
Secondary	0.29 (−1.7, 2.3)	0.800	2.1 (0.16, 4.1)	0.034
High school	0.78 (−1.3, 2.9)	0.500	1.6 (−0.48, 3.6)	0.130
University/postgrad	0.73 (−1.5, 3.0)	0.500	2.1 (−0.11, 4.3)	0.062
**BMI (kg/m^2^)**	−0.01 (−0.14, 0.13)	0.900	−0.15 (−0.28, −0.02)	0.029
**Nutritional knowledge (reference: poor)**	1	–	–	–
Good	−0.20 (−0.92, 0.52)	0.580	−0.35 (−0.88, 0.18)	0.200

Multiple linear regression, adjusting for age, gender, income, education, and BMI.

Among demographic and clinical covariates, BMI was significantly associated with lower HbA1c (β = −0.15; 95% CI: −0.28, −0.02; *P* = 0.029), a finding that warrants cautious interpretation due to its counterintuitive direction. Age was positively associated with HbA1c (β = 0.03; 95% CI: 0.00, 0.06; *P* = 0.041), while secondary education level was also associated with higher HbA1c (β = 2.1; 95% CI: 0.16, 4.1; *P* = 0.034) compared to primary education. Gender, income, and fiber intake showed no significant associations with HbA1c or glucose (all *P* > 0.05). Participants with good nutritional knowledge had lower HbA1c values on average (β = −0.35), though this was not statistically significant (*p* = 0.20).

Moderation analysis indicated that nutritional knowledge did not significantly moderate the relationship between dietary intake (energy, carbohydrates, fats) and metabolic parameters (all interaction *p*-values > 0.05), suggesting that knowledge alone is insufficient without behavior-focused interventions.

Multivariable logistic regression model examining predictors of poor nutritional knowledge and poor dietary behavior, presented as odds ratios (OR), 95% CI, and *p*-values (Table 5). In adjusted analyses, higher BMI was significantly associated with a lower likelihood of poor nutritional knowledge and poor dietary behavior (OR = 0.70; 95% CI: 0.54, 0.88; *P* = 0.003 for both outcomes). This finding suggests that individuals with higher BMI in this sample were more likely to possess better nutritional knowledge and engage in healthier dietary practices, though this may reflect reverse causality or confounding (e.g., prior counseling due to weight concerns). No significant associations were observed for age, gender, or income level in relation to either poor knowledge or behavior (all *P* > 0.05). Participants with secondary or high school education showed higher odds of poor nutritional knowledge and behavior compared to those with only primary education; however, these associations were not statistically significant and were marked by extremely wide confidence intervals (e.g., OR for secondary education = 5.29; 95% CI: 0.16, 179; *P* = 0.3), indicating substantial uncertainty and potential model instability. For participants with university or postgraduate education, estimates could not be calculated due to lack of data variation (OR = 0.00; NA), suggesting sparse or missing cases in this subgroup.

**TABLE 5:** Multivariable regression model (split for nutrition status subgroups).

Characteristic	Poor nutritional knowledge (OR, 95% CI)	*p*-value	Poor dietary behavior (OR, 95% CI)	*p*-value
**Age (years)**	0.99 (0.93, 1.05)	0.700	0.99 (0.93, 1.05)	0.700
**Gender (reference: male)**	1	–	–	–
Female	1.07 (0.23, 4.41)	0.900	1.07 (0.23, 4.41)	0.900
**Income (reference: < 5 million VND)**	1	–	–	–
≥ 5 million VND	1.23 (0.30, 4.99)	0.800	1.23 (0.30, 4.99)	0.800
**Education (reference: primary)**	1	–	–	–
Secondary	5.29 (0.16, 179)	0.300	5.29 (0.16, 179)	0.300
High school	2.18 (0.06, 83.2)	0.600	2.18 (0.06, 83.2)	0.600
University/postgrad	0.00 (NA, NA)	0.900	0.00 (NA, NA)	0.900
**BMI (kg/m^2^)**	0.70 (0.54, 0.88)	0.003	0.70 (0.54, 0.88)	0.003

Logistic regression, adjusting for age, gender, income, education, and BMI.

## 5 Discussion

This study examined the 24-h dietary intake and its relationship with nutritional knowledge and behaviors among older adults with type 2 diabetes mellitus. The findings reveal significant differences in dietary intake, nutritional knowledge, and dietary behaviors across different nutritional status groups. Overweight/obese participants exhibited higher energy, protein, carbohydrate, and fat intake than normal and underweight groups. Poor nutritional knowledge and dietary behaviors were most prevalent among overweight/obese individuals (51%), indicating a critical gap in diabetes management practices. Additionally, increased energy intake was associated with higher HbA1c levels (*P* = 0.049), while carbohydrate intake showed an inverse relationship with HbA1c (*P* = 0.049). Higher BMI was correlated with lower HbA1c levels (*p* = 0.029), suggesting a complex interplay between body composition and glycemic control. Older adults with T2DM face unique barriers to dietary management, including limited access to nutritional education, economic constraints, and age-related challenges such as reduced appetite and difficulty integrating dietary changes (2). These barriers, particularly prevalent among overweight/obese individuals (51% with poor nutritional knowledge), underscore the need for targeted interventions to address knowledge gaps and socioeconomic limitations in this population. The inverse relationship between carbohydrate intake and HbA1c (β = −0.60, *P* = 0.049) was unexpected, as higher carbohydrate intake typically correlates with elevated HbA1c. This may reflect higher consumption of fiber-rich carbohydrates (e.g., fruits, vegetables) rather than refined sugars, as Vietnamese diets often include fruit-heavy patterns (4). However, the lack of specific analysis on total sugar intake limits our ability to differentiate carbohydrate sources, a limitation for future research. Additionally, underreporting of carbohydrate intake, common among older adults and T2DM patients due to recall bias or social desirability, may have influenced these findings (12).

Our findings align with prior research emphasizing the role of dietary intake in diabetes management. Razaz et al. (8) showed that medical nutrition therapy reduced HbA1c by 0.43% and weight by 1.54 kg, supporting the need for structured dietary interventions. In Vietnam, where 60% of T2DM patients have poor glycemic control (3), and only 16.3% adhere to diets (2), culturally tailored nutrition education, as recommended by Evert et al. (14), could address high-carbohydrate intake (168.9–199.2 g/day) by promoting balanced diets. Abdullah et al. (15) highlighted that dietary counseling in family practice significantly improves diabetes outcomes. Their study reported that integrating structured dietary counseling in primary care settings reduced HbA1c by an average of 0.5% over six months, demonstrating the effectiveness of personalized nutrition interventions. This aligns with our findings, which show a significant correlation between energy intake and HbA1c levels. However, despite these improvements, dietary adherence remains a challenge, particularly among overweight/obese individuals who continue to consume excessive calories and macronutrients.

The PANDA trial further supports this notion, showing that patients with T2DM often exceed recommended intake levels (16). Their study found that after a three-month dietary intervention, daily sodium intake was reduced by 561 mg, saturated fat intake decreased by 2.9 g, and added sugar consumption dropped by 7 g (*P* < 0.050 for all). Despite these improvements, the majority of participants still exceeded dietary guidelines. This underscores the difficulty in achieving sustained dietary modifications, which is consistent with our study’s findings that overweight/obese individuals struggle with dietary adherence.

The role of diet in glycemic control is well-documented in prior studies. Setianto et al. (17) reported that patients with regular dietary habits had significantly lower random blood glucose (RBG) levels (*P* = 0.000), reinforcing our results that increased energy intake is correlated with higher HbA1c. Additionally, Kimura et al. (18) demonstrated that higher fiber intake was associated with a 47% reduced risk of developing diabetes (HR = 0.53, 95% CI: 0.31–0.90) (6). Although our study did not find a significant association between fiber intake and glycemic markers, these findings suggest that fiber consumption may be protective in diabetes management.

Micronutrient intake and diabetes risk remain a debated topic. Eshak et al. (19) found that higher vitamin K intake was associated with a 29% reduced risk of developing T2DM (OR = 0.71, 95% CI: 0.54–0.93, *p*-trend = 0.01). Conversely, Pang et al. (20) reported that elevated serum retinol levels increased diabetes risk by 113% (OR = 2.134, 95% CI: 1.377–3.306, *P* = 0.009). In our study, we did not observe a clear relationship between vitamin intake and glycemic markers, suggesting that further investigation is needed to determine the role of micronutrients in diabetes progression in Vietnamese populations.

Public health policies addressing dietary intake can have substantial impacts on diabetes management. Hasenegger et al. (21) emphasized that processed foods contributed 75% of total dietary sodium intake in Austria. Given the growing consumption of processed foods in Vietnam, regulatory measures, such as improved food labeling and sodium reduction strategies, may help mitigate the impact of unhealthy dietary habits. Similarly, López-Olmedo et al. (22) found that Mexican adults, regardless of diabetes status, had low adherence to nutritional guidelines, with an average score below 50 points on the Mexican Alternate Healthy Eating Index (MxAHEI). These findings highlight the global challenge of poor dietary adherence among diabetes patients. International evidence supports structured nutrition education as a cornerstone of T2DM management. The ADA recommends DSMES programs that include nutrition therapy to enhance self-efficacy and adherence, with global studies showing HbA1c reductions of 0.3–1.0% (7, 14). In Vietnam, where 51% of overweight/obese patients exhibit poor nutritional knowledge, integrating such programs with tools like the Vietnamese DSCKQ-30 could address knowledge gaps and improve dietary adherence (23).

Cultural and economic factors also play a significant role in dietary behaviors. Ilunga Tshiswaka et al. (24) underscored how cultural perceptions shape dietary choices among Congolese immigrants, emphasizing the need for culturally tailored interventions. In Vietnam, high-carbohydrate diets (168.9–199.2 g/day) reflect rice-based cultural norms, similar to patterns in Bui et al. (4). Compared to the general population’s 2,000 kcal/day and 65–70% carbohydrate intake (4), T2DM patients’ lower energy (1,104.9–1,331.1 kcal/day) suggests restrictive diets or under-reporting. Interventions, per Evert et al. (14), could adapt rice dishes with whole grains to align with T2DM needs. Similarly, Leone et al. (25) reported that Saharawi women with higher adherence to unhealthy dietary patterns exhibited significantly higher insulin resistance (b = 2.49, 95%, CI: 0.41–4.57, *P* = 0.02). These findings suggest that dietary interventions in Vietnam must consider cultural and nutritional preferences to ensure their effectiveness and sustainability.

The impact of dietary patterns on inflammation and insulin resistance further supports the need for dietary modifications. Jin et al. (5) found that individuals consuming hyperinsulinemic and proinflammatory nutritional patterns had a 49% increased risk of developing diabetes (HR = 1.49, 95% CI: 1.32–1.68, *p*-trend < 0.0001). This aligns with our study’s findings that increased energy intake was associated with higher HbA1c levels, reinforcing that dietary patterns influence metabolic outcomes.

Moreover, social determinants of dietary behavior must be considered. Kurnia et al. (26) found that social support significantly predicted improved dietary behavior (β = 0.272, *P* < 0.001). This highlights the importance of community-based interventions encouraging family and peer support in diabetes management. Similarly, Loubna et al. (27) emphasized that lack of dietary knowledge contributes to poor adherence, reinforcing our finding that overweight/obese individuals exhibited lower levels of nutritional knowledge and dietary behavior adherence.

The role of dietary management behaviors in diabetes outcomes has been further emphasized by Liu et al. (6), who reported that 78% of Chinese diabetic patients with dietary management behaviors had higher diet quality scores than those without structured dietary management. This suggests that structured dietary education programs can significantly improve adherence and glycemic control, supporting our recommendation for increased nutrition education efforts in Vietnam.

Finally, dietary improvements have long-term health benefits. Ley et al. (28) found that a > 10% improvement in diet quality over four years was associated with a 16% reduced risk of developing diabetes (HR = 0.84, 95% CI: 0.78–0.90). Conversely, a > 10% decrease in diet quality was linked to a 34% higher diabetes risk (HR = 1.34, 95% CI: 1.23–1.46). These findings reinforce the importance of continuous dietary monitoring and intervention to prevent diabetes progression.

Overall, our study contributes to the growing evidence that dietary intake plays a crucial role in diabetes management. The findings underscore the need for improved dietary counseling, culturally appropriate nutrition interventions, and public health policies to reduce unhealthy food consumption. Given the high carbohydrate intake (199.2 g/day in overweight/obese patients) and low adherence (16.3%), (2) results can guide monthly dietary counseling programs, as effective in Tran et al. (2) (AOR = 3.01), focusing on portion control and low-glycemic foods. Community-based education, leveraging tools like the DSCKQ-30, (23) can address the 51% poor knowledge rate in overweight/obese patients. Policies promoting healthier food access, as suggested by Bui et al. (4), could mitigate socioeconomic barriers. By integrating these approaches, diabetes management strategies can more effectively promote long-term health outcomes for individuals with T2DM in Vietnam.

### 5.1 Limitations

This study has several limitations. First, potential underreporting of dietary intake, particularly carbohydrates, may have affected the observed inverse relationship with HbA1c, as older adults and T2DM patients often underreport due to memory issues or social pressures. Second, the absence of total sugar intake analysis limits insights into carbohydrate quality. Future studies should consider including total sugar as an independent dietary variable in modeling HbA1c outcomes. Third, the nutritional knowledge and dietary behavior questionnaires were not formally validated, though developed with expert input. Additionally, the sample size (*n* = 355) is relatively small for a high-prevalence condition like T2DM, which may limit statistical power and the reliability of results. Moreover, the study population was drawn from patients receiving care at a single medical center, which may introduce selection bias and limit generalizability to the broader Vietnamese T2DM population or those without access to care. Additionally, Bonferroni correction for multiple testing in the regression model may be conservative due to correlated predictors, potentially reducing power and rendering borderline associations non-significant; however, it was applied to all tests to rigorously control Family-Wise Error Rate (FWER). Finally, the cross-sectional design precludes causal inferences.

### 5.2 Implications for practice

The findings have important implications for diabetes management and public health policy in Vietnam. Given the significant association between dietary intake and glycemic control, integrating structured dietary counseling programs in primary care settings is essential. As Abdullah et al. (15) recommended, incorporating dietitians into healthcare teams can enhance patient education and adherence to dietary guidelines. Their study found that patients receiving structured dietary counseling had an 18% greater adherence to recommended dietary guidelines compared to those who did not.

Furthermore, tailored dietary interventions targeting specific macronutrient intake should be developed. The PANDA trial showed that structured meal plans effectively reduced sodium, fat, and sugar intake among diabetes patients (16). Adopting a similar approach in Vietnam could improve dietary adherence and health outcomes. Liu et al. (6) found that 78% of Chinese diabetic patients with dietary management behaviors had higher diet quality scores than those without, further supporting the need for structured nutritional interventions.

### 5.3 Future research directions

Future research should explore the longitudinal impact of dietary behaviors on diabetes progression. Investigating culturally tailored interventions to improve nutritional knowledge and adherence is also warranted. Additionally, examining the role of micronutrient intake, particularly fat-soluble vitamins and trace elements, in diabetes management could provide deeper insights.

Expanding research to include qualitative studies on barriers to dietary adherence may enhance intervention strategies, as Loubna et al. (27) suggested. Understanding sociocultural influences, such as family support and economic constraints, can inform more effective policies for diabetes dietary management in Vietnam. Kurnia et al. (26) found that social support was a significant predictor of improved dietary behavior (β = 0.272, *p* < 0.001), suggesting that community-based interventions may enhance dietary adherence.

## 6 Conclusion

Older adults with T2DM in Vietnam exhibit significant variations in dietary intake, with poor nutritional knowledge and low dietary adherence, particularly among overweight/obese individuals. Barriers such as limited education, economic constraints, and age-related challenges underscore the need for targeted interventions. Structured nutritional counseling, culturally tailored education, and public health policies (e.g., improved food labeling) could enhance glycemic control and address these gaps, particularly for older adults.

## Data Availability

The raw data supporting the conclusions of this article will be made available by the authors, without undue reservation.
